# Antigen-specific influence of GM/KM allotypes on IgG isotypes and association of GM allotypes with susceptibility to *Plasmodium falciparum *malaria

**DOI:** 10.1186/1475-2875-8-306

**Published:** 2009-12-22

**Authors:** Hayder A Giha, Amre Nasr, Nnaemeka C Iriemenam, David Arnot, Marita Troye-Blomberg, Thor G Theander, Klavs Berzins, Gehad ElGhazali, Janardan P Pandey

**Affiliations:** 1 Department of Biochemistry, Faculty of Medicine and Medical Sciences, Arabian Gulf University (AGU), PO Box 26671, Manama, Kingdom of Bahrain; 2 Malaria Research Center (MalRC), Department of Biochemistry and Department of Microbiology and Immunology, Faculty of Medicine, University of Khartoum, PO Box: 102, Khartoum, Sudan; 3 Department of Immunology, Wenner-Gren Institute, Stockholm University, Stockholm, Sweden; 4 Tropical Diseases Research Laboratory, Department of Medical Microbiology & Parasitology, College of Medicine, University of Lagos, PMB 12003 Lagos, Nigeria; 5 Centre for Medical Parasitology at Department of International Health, Immunology and Microbiology, University of Copenhagen and Department of Infectious Diseases, Copenhagen University Hospital, Copenhagen, Denmark; 6 Faculty of Medicine, King Fahad Medical City, Riyadh, Saudi Arabia; 7 Department of Microbiology and Immunology, Medical University of South Carolina, Charleston, SC 29425, USA

## Abstract

**Background:**

*Plasmodium falciparum *malaria is a complex disease in which genetic and environmental factors influence susceptibility. IgG isotypes are in part genetically controlled, and GM/KM allotypes are believed to be involved in this control.

**Methods:**

In this study, 216 individuals from Daraweesh, an area of seasonal malaria transmission in Sudan, were followed for nine years for malaria infection. Total IgG and IgG isotypes against four malaria antigens, MSP2-3D7, MSP2-FC27, AMA1, and Pf332-C231 were measured in plasma obtained from the cohort at the end of the study, during the dry malaria-free period. The GM/KM allotypes of the donors were determined.

**Results:**

The GM 1,17 5,13,14,6 phenotype was associated with a higher incidence of malaria compared with the non-1,17 5,13,14,6 phenotypes (P = 0.037). Paradoxically, the carriers of the GM 1,17 5,13,14,6 phenotype had significantly higher baseline levels of total IgG and non-cytophilic IgG isotypes as compared to non-carriers. The KM allotypes influence on IgG isotypes level was limited. Finally, the differences in the baseline concentrations of total IgG and IgG isotypes between the different GK/KM phenotype carriers were antigen-dependent.

**Discussion:**

The results show that GM but not KM allotypes appeared to influence host susceptibility to uncomplicated malaria as well as the antibody profile of the donors, and the carriers of the GM 1,17 5,13,14,6 phenotype were the most susceptible

**Conclusions:**

The GM allotypes have significant influence on susceptibility to uncomplicated *P. falciparum *malaria and antigen-dependent influence on total IgG and IgG subclasses.

## Background

Selection for resistance to malaria among inhabitants of malaria endemic regions may have influenced polymorphisms in genes encoding a variety of proteins involved in immunity [1]. For example, different subclasses of immunoglobulin G (IgG isotypes) have been proposed to play opposing roles in protection against malaria [2]. Cytophilic IgG (IgG1 and IgG3) antibodies were shown to be protective, while non-cytopihlic ones (IgG2 and IgG4) were found to be competing with the former isotypes [2,3]. Thus, not only levels, but also switching between IgG isotypes is believed to play a role in development of protective immunity. Protein polymorphism within the individual IgG subclasses is in part due to GM/KM allotypes, which are genetically determined serologically detectable antigenic determinants. These allotypic determinants are expressed on both the heavy and light chains of IgG1, IgG2, and IgG3. The combination of individual alleles is referred to as a haplotype [4] and GM haplotypes vary among ethnic groups [5]. KM gene frequencies also vary significantly among various ethnic groups. However, the deployment of GM/KM allotyping for human population genetic analysis, mapping global haplotype distributions, indicated that selection on GM haplotypes is low at the human population level [6]. It has also been reported that the levels of the IgG subclasses are influenced by the GM allotypes in adult Caucasian blood donors [7] and in African American populations [8].

The association of GM/KM allotypes with susceptibility to several different diseases has been reported [9] and their involvement in autoimmune disease has also been proposed [10]. Some data have also indicated a possible association of GM/KM allotypes with malaria morbidity and severity [11]. Differences between ethnic groups in the distribution of GM/KM allotypes and a possible association with malaria susceptibility were recently demonstrated in a study carried out in eastern Sudan involving comparison of groups of West African Fulani origin with indigenous sympatric tribes [12]. At present, there is limited evidence for the involvement of human IgG allotypes leading to functional differences in IgG antibodies as compared to the evident differences seen between IgG subclasses in malaria [2].

In the current study, a hypothesis suggesting that the GM/KM make-up of individual immunoglobulin affects IgG isotype levels, depending on target malaria antigen, was examined. Consequently, the GM/KM allotypes might influence the host susceptibility to malaria. Therefore, ten GM (1, 2, 3, 5, 6, 13, 14, 17, 21, 23) and 2 KM (1, 3) allotypes were investigated and combined with nine years of longitudinal malaria incidence data collection. In addition, baseline antibody response to four leading asexual blood-stage malaria vaccine-candidate antigens, comprising the apical membrane antigen-1 (AMA-1), merozoite surface protein-2 (MSP-2; 3D7 and FC27 alleles), and Pf332-C231, was analysed to test this hypothesis. Results revealed that, development of protective immunity is not only attributed to repeated exposure with increasing age [13], but also to genetic polymorphisms of the IgG in terms of GM/KM phenotypes.

## Methods

### Study area

The study was carried out in Daraweesh village, eastern Sudan, where malaria is hypoendemic with occasional quite severe 'malaria seasons'. The malaria transmission in the region is strictly seasonal but markedly unstable; in 'wet years' it peaks in October/November, after the summer rain, although a few sporadic cases also occur between February and August. In Daraweesh, malaria affects all age groups, although the incidence decreases after twenty years of age. A detailed geographical, demographic and social description of the area has previously been reported [14,15].

### Study population

The inhabitants of Daraweesh are descendents of a founder population of the West African Fulani-speaking group, originally from Burkina Faso. The village founders migrated to the Sudan more than hundred years ago although the ethnic identity (and Fulani language) has been maintained by frequent inter-marriage between descendents of the small founding group and some marriage with other Sudanese Fulani immigrants. In year 2000, the total population was approximately 500 inhabitants and during the 13-year study period, it ranged between, 400 to 600 individuals. The plasma samples used in this study were collected in the dry season in May 2005. All donors were malaria-free, no malaria parasite was detected by microscopy or PCR in any of the blood samples.

### Longitudinal clinical and parasitological surveillances

From 1991 to 2004, the inhabitants of Daraweesh were under regular clinical and parasitological malaria surveillance [14,15]; except for four malaria seasons, where funds were not available or follow-up was not complete. Malaria was monitored by active and passive surveillances by a research team over this period. The malaria was diagnosed if a patient had fever (oral temperature >37.5 °C), or reported fever, and had microscopically-detectable parasitaemia after examination of a maximum of 200 fields under oil immersion on each slide. Malaria patients were treated with chloroquine or with pyrimethamine/sulphadoxine in case of chloroquine treatment failure or by a combination of both as in year 2003 (therapeutic trial). Notably, severe malaria incidence was extremely low during the entire study period. Although prophylaxis was not used, presumptive treatment was sometimes practiced.

### Blood samples

Peripheral blood samples (3 ml) were collected from each donor into a vacuum EDTA tube. After centrifugation the plasma was collected into cryotubes and stored at -20°C until used. The study had received ethical clearance from the ethical committee at the Faculty of Medicine, University of Khartoum, and the Sudanese Federal Ministry of Health. Consent was obtained from the village members regarding the whole project and from the individual donors, when blood samples were collected.

### Measurement of total IgG and IgG isotypes by enzyme-linked immunosorbent assay (ELISA)

An indirect enzyme-linked immunosorbent assay, ELISA, [16] was used for measurement of total IgG and the subclasses; IgG1, IgG2, IgG3 and IgG4, against four asexual blood-stage antigens; MSP2-3D7, MSP2-FC27, AMA-1 and Pf332-C231 [17]. Briefly, EIA/RIA plates (Costar, MA, USA) were coated with AMA-1 at 1 μg/ml, 3D7 MSP 2 and FC27 MSP 2 at 1 μg/ml, and Pf332-C231 at 5 μg/ml. The AMA-1 protein, which has an N-terminal hexa-His tag, was expressed in *Escherichia coli *and refolded *in vitro*. Both the 3D7 and FC27 forms of MSP 2 were expressed in *E. coli *with C-terminal hexa-His tags. The expression and purification of these proteins will be described elsewhere (manuscript in preparation). The recombinant Pf332-C231 corresponds to a 231-amino acid fragment in the C-terminal part of Pf332. The plates were incubated overnight at 4°C, and then blocked for 2 hrs with 0.5% bovine serum albumin (BSA) diluted in carbonate buffer (pH 9.6). Plasma samples diluted in incubation buffer (PBS + 0.5% BSA), 1:1,000 (IgG) and 1:400 (IgG1-4), were added in duplicate and incubated for 1 h at 37°C. The plates were then washed four times, and bound IgG antibodies were detected with goat anti-human IgG-ALP (1:2000) (Mabtech, Nacka, Sweden). IgG subclasses were analysed with their respective biotin conjugated mouse anti-human subclass specific monoclonal antibodies: mouse anti-human IgG1 1:1,000 (M15015, Clone NL16, SkyBio, Bedfordshire, UK), mouse anti-human IgG2 1:3,000 (555874, Pharmingen, Erembodegem, Belgium), mouse anti-human IgG3 1:1,000 (MH 1532, Caltag laboratories, Paisley, UK) and mouse anti-human IgG4 1:2000 (B3648, Sigma, St. Louis, USA). Alkaline phosphatase (ALP) conjugated streptavidin (Mabtech) diluted 1:2,000 was added to detect bound antibodies of IgG2-4, while ALP-conjugated to goat anti-mouse Ig (Dakopatts, Glostrup, Denmark; 1:1,000) were used for IgG1 antibodies and the plates were developed with nitrophenyl phosphate (Sigma-Aldrich Chemie GmbH, Steinheim, Germany). The absorbance was read at 405 nm using a Vmax™ Kinetic microplate reader (Molecular devices, Menlo Park, USA) and the antibody concentrations were deduced from the log-log correlative coefficient of each IgG subclass standard curve (six dilutions of myeloma proteins of IgG1-4 subclasses), ranging from 0.01 to 3 μg/ml for IgG1, 0.001 to 0.3 μg/ml for IgG2, 0.001 to 0.1 μg/ml for IgG3 and 0.01 to 1 μg/ml for IgG4 according to the manufacturer's recommendation (Biogenesis, Poole, England). In plots, the antibody concentrations were transformed into log 10 values without any changes in the P - values obtained from the analysis of the raw values.

### Determination of GM and KM allotypes

Allotyping of the serum samples from the above subjects was carried out for G1M (1,2,3,17), G2M (23), G3M (5,6, 13,14,21), and KM (1,3) determinants by a standard haemagglutination-inhibition method [18]. Human erythrocytes (Group O Rh+) coated with anti-Rhesus antibodies of known GM allotypes and mono-specific anti-allotype sera were employed. Sera containing IgG of particular allotype inhibit haemaggultination, but negative sera do not.

### Statistical analysis

Sigma Stat software was used for data analysis. There is almost absolute linkage disequilibrium between individual GM alleles [19]; therefore, data were analysed as haplotypes and also according to presence or absence of individual markers. For comparisons of measurable parameters e.g. age, levels of antibodies and etc, between different categories grouped on basis of GM or KM allotyping, ANOVA or Kruskal-Wallis One Way Analysis of Variance on Ranks was used. For pair wise analysis of the same parameters between the same groups of donors, T-test or Mann-Whitney Rank Sum Test was used.

## Results

### Frequency of GM and KM allotypes in Daraweesh

The individual GM allelic type (allotype) 5 and 17 were detected in all (100%) donors involved in this study. The vast majority of the donors had the GM 1 (99.5%) and GM 14 (98.6%) alleles. The GM 13 (82.4%) allotype was the next most frequent, while half of the population carried the GM 6 allotype (49.5%). The other allotypes; 3, 21 and 23, were rare (Table 1). The donors were categorized into five groups based on their GM phenotypes (combination of allotypes), four predominant phenotypes and a fifth group which combines six minor groups taken together and referred to as "others". Most of the donors were carrying the four most frequent phenotypes; 1,17 5,13,14 (40.3%) and 1,17 5,13,14,6 (38.4%), followed by 1,17 5,14 (9.3%) and 1,17 5,14,6 (6.9%), and the "other" rare phenotypes were altogether accounting for 5.1%, (Table 2). The frequencies of the KM 1, 3, and 1,3 phenotypes were; 8.3%, 52.8% and 38.9%, respectively. The age distribution of both GM and KM allotypes were comparable between all study groups. The majority (65.7%) of the donors were females (Table 2).

**Table 1:** The frequency of the individual GM allotypes of IgG immunoglobulin in Daraweesh village.

GM allotypes	Proportions (number of individuals)
5	100% (216)
17	100% (216)
1	99.5% (215)
14	98.6% (213)
13	82.4% (178)
6	49.5% (107)
3	1.9% (4)
21	1.4% (3)
23	0.5% (1)

**Table 2:** The frequency of the GM phenotypes (upper rows) and KM allotypes (bottom rows) of IgG immunoglobulin in Daraweesh village.

GM/KM Phenotypes	Frequency	Age (years)	Sex (F/M)
**GM**		Mean ± SD	
1,17 5,14	9.3% (20)	20.2 ± 8.341	18/2
1,17 5,14,6	6.9% (15)	22.9 ± 16.1	8/7
1,17 5,13,14	40.3% (87)	26.2 ± 17.8	61/26
1,17 5,13,14,6	38.4% (83)	26.8 ± 17.0	48/35
Others [n, 6]	5.1% (11)	22.9 ± 16.9	7/4
		P = 0.324	
**KM**			
1	8.3% (18)	23.4 ± 13.1	15/3
3	52.8% (114)	24.9 ± 17.2	67/47
1,3	38.9% (84)	26.8 ± 18.4	60/24
	Kruskal-Wallis	P = 0.872	
Total	216		142/74

The age and sex of the donors are shown.

The others are: 1,17 5,6 = **3**; 17 5,13,14 = **1**; 1,3,17 5,13,14 = **1**; 1,3,17 5,13,14,6 = **2**; 1,17 5,13,14,6,21 = **3**; 1,3,17 23 5,13,14,6 = **1**

### Association of IgG allotypes with susceptibility to malaria

The average number of previous malaria episodes experienced by each of the donors over nine years was used as an index for differential susceptibility to malaria between the GM or KM allotype groups. The GM 1,17 5,13,14,6 phenotype carriers experienced the highest number of malaria episodes over the nine years of the follow up (mean ± SD, 1.9 ± 1.5, range 0-7), followed by 1,17 5,14,6 (1.5 ± 1.6) and 1,17 5,13,14 (1.5 ± 1.3) phenotype carriers, (Table 3). The lowest number of malaria episodes was experienced by carriers of phenotype 1,17 5,14 (1.3 ± 1.5) and the carriers of the minor allotypes (1.4 ± 1.6). Taken all together, differences in the number of malaria episodes between all GM phenotypes were not statistically detectable, P = 0.273, Kruskal-Wallis one way analysis of variance on ranks.

**Table 3:** The mean number and range of previous malaria episodes experienced by donors with different GM/KM allotypes/phenotypes, over 9-years of follow up in Daraweesh

Donors grouping	Number of malaria episodes		P-values
		
	Mean ± SD	Range	
**GM allotypes**			
1,17 5,14	1.3 ± 1.5	0 - 5	P = 0.273 Kruskal-Wallis One Way Analysis of Variance on Ranks
1,17 5,14,6	1.5 ± 1.6	0 - 5	
1,17 5,13,14	1.5 ± 1.3	0 - 4	
1,17 5,13,14,6	1.9 ± 1.5	0 - 7	

**KM allotypes**			
1 (18)	1.7 ± 1.2	0 - 3	P = 0.342 Kruskal-Wallis
3 (114)	1.7 ± 1.5	0 - 7	
1,3 (84)	1.5 ± 1.4	0 - 6	
			

**Figure 1:**
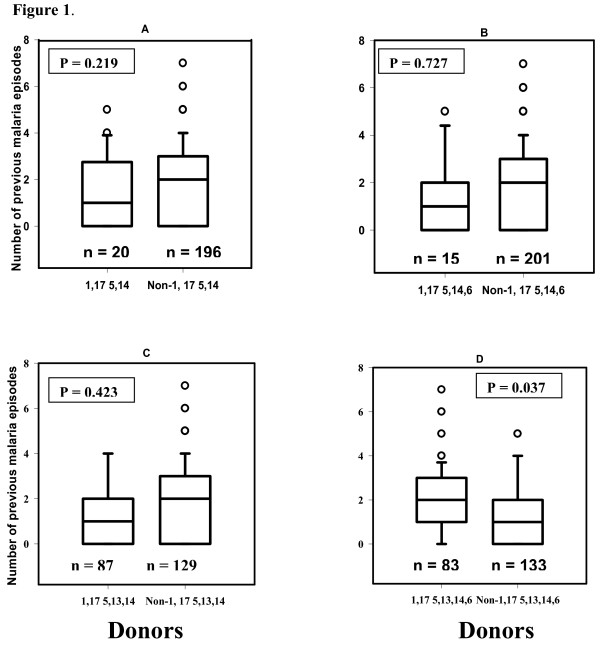
**The figure shows the average number of malaria episodes experienced by the carriers of the major GM phenotypes; 1,17 5,14 (a) - 1,17 5,14,6 (b) - 1,17 5,13,14 (c) - 1,17 5,13,14,6 (d), during nine years of follow up in Daraweesh village**. Comparing the carriers versus non-carrier of each of the 4 phenotypes, the only significant difference was that of 1,17 5,13,14,6 (d). The box represents 25-75 percentile, the horizontal line is the median value vertical bar is 95 percentile and open circle are the outliers

However, grouping of the donors based on presence and absence of certain set of GM allotypes (phenotypes), showed that carriers of 1,17 5,13,14,6 phenotype as compared to the non-carriers, had experienced statistically discernibly increased number of malaria episodes over the years of the follow up (P = 0.037). The carriers of the other three major GM phenotypes as compared with the non-carriers of the corresponding phenotypes were equally susceptible to malaria (Figure 1). To test whether an individual allotype was associated with increased susceptibility, the analysis was repeated comparing the allotype 6 (1.8 ± 1.5, episodes) versus non-6 allotype (1.5 ± 1.4) carriers, P = 0.148; and allotype 13 (1.7 ± 1.4) versus non-13 allotype (1.3 ± 1.5) carriers, (P = 0.123). The other individual allotypes were either present in almost all combinations e.g. allotype 17, 5, and 1 and 14, or were very rare e.g. allotypes 3, 21 and 23. However, the KM allotype carriers experienced a comparable number of malaria episodes during the longitudinal follow up, whether the comparison was made between the three KM allotype carriers; 1, 3 and 1,3, together or in pairs (Table 3).

### Total IgG and IgG isotype concentrations in relation to GM allotypes

**Figure 2:**
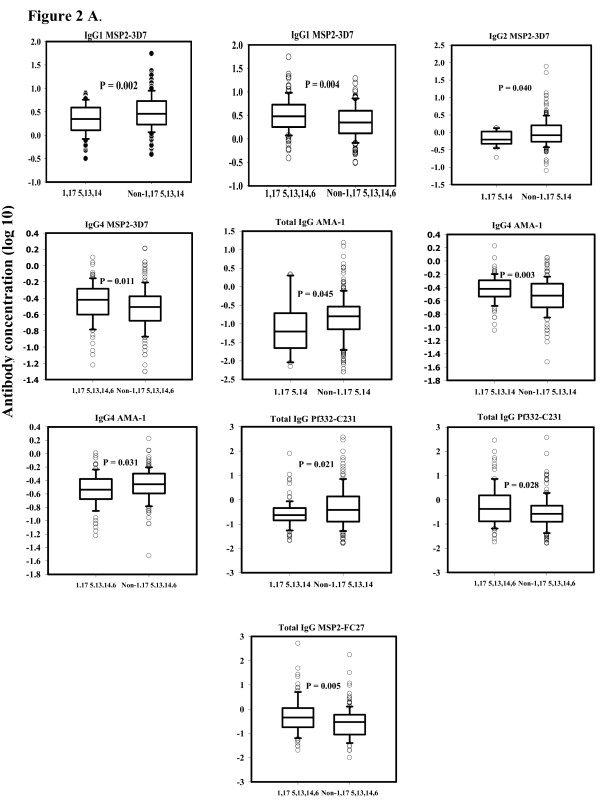
**Concentrations (log 10) of total IgG and IgG isotypes to four antigens: MSP2-3D7, AMA-1, Pf332-C231 and MSP2-FC27**. The figure demonstrates the significant differences in levels of the above antibodies between the each of the GM phenotype carriers; 1,17 5,14 - 1,17 5,14,6 - 1,17 5,13,14 - 1,17 5,13,14,6, and the corresponding-phenotype non-carriers.

The levels of the total IgG and IgG isotypes against the four tested malaria antigens showed marked variation between the isotypes, and also for the same isotype between the antigens. For a specific IgG isotype, the concentration varies between individuals with different GM allotypes, depending principally on the target antigen. Comparing the carriers of specific phenotype with the non-carriers of that phenotype was sometimes statistically significant. The carriers of the 1,17 5,13 14,6 phenotype had the highest frequency of high levels of IgG and IgG isotypes in their sera, followed by the carriers of the 1,17 5,13,14 and 1,17 5, 14. However, there was no significant difference between the carriers and non-carriers of the 1,17 5, 14,6 phenotype (Figure 2).

### Total IgG and IgG isotype concentrations in relation to KM allotypes

**Figure 3:**
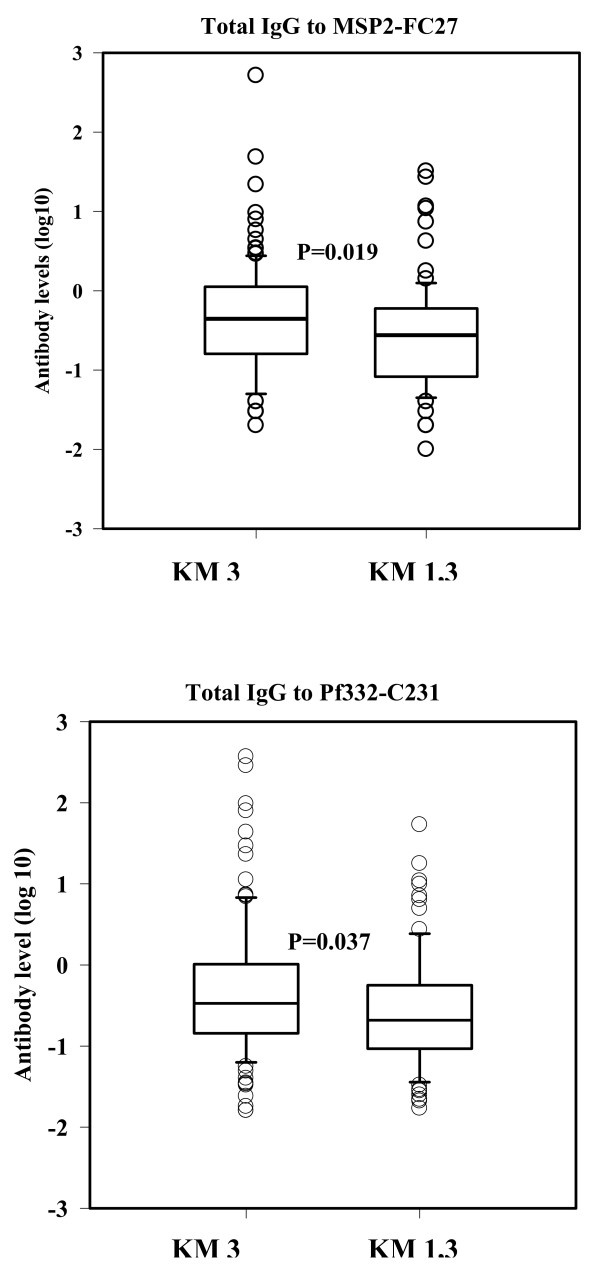
**Concentrations (log 10) of total IgG and IgG isotypes to two antigens: Pf332-C231 and MSP2-FC27**. The figure demonstrates the significant differences in levels of the above antibodies between the each of the KM 1, 1,3 and 3 allotype carriers. The box represents 25-75 percentile, the horizontal line is the median value vertical bar is 95 percentile and open circle are the outliers. Only comparisons of statistically significant differences are presented

The above comparisons of the IgG and its isotype concentrations to the four target antigens between allotypes carriers were repeated, but using KM (1, 3, and 1,3) instead of GM allotypes. Results showed that there was less influence of the KM allotype on the total IgG and IgG isotypes concentration, regardless of the target antigen. The only significant differences were the higher concentration of total IgG to MSP2-FC27 and to Pf332-C231 antigens in the plasma obtained from the KM 3 compared to that obtained from the KM 1,3 phenotype carriers (Figure 3).

## Discussion

In malaria hyper-endemic settings, protection from malaria mortality and morbidity is achieved with age as a consequence of immunity and this protection is augmented in subpopulations by inherent, genetically controlled host factors. The genetic factors involved in resistance to malaria are clearly numerous, the best known involving various balanced polymorphisms in haemoglobin genes. In this study, the number of malaria episodes over nine years of clinical and parasitological follow-up was recorded for a cohort of around half of the Daraweesh village inhabitants. The data showed who was most susceptible/resistant to malaria infection. Integration of this data together with genotyping of GM/KM allotypes and measurement of total IgG and IgG isotypes to four malaria antigens revealed interesting findings. The study showed that, the GM allelic combinations but not the KM allelic combinations had significant influence in uncomplicated malaria susceptibly and in the type and concentration of IgG isotype. While the implication of GM allotypes in susceptibility to bacterial infections and other diseases have been known for some time [20,21], the role of GM/KM polymorphisms in malaria susceptibility has only recently been investigated [12,22]. In this study, the GM 1,17 5,13,14,6 phenotype was significantly associated with increased susceptibility to malaria infections although paradoxically it was significantly associated with the highest baseline concentration of certain IgG and IgG isotypes. Both associations were age independent, and the latter one was largely dependent on the target antigen.

GM allotypes have been assessed in all villagers tested and the frequencies of each GM or KM allotype determined. Nearly all villagers have GM allotypes 5, 17, 1 and 14 in their GM phenotype. This leaves allotypes 13 and 6 as the major GM allotype differences between villagers, both of which are located within the G3 constant region. Allotypes 1 and 17 are believed to be located in the G1 constant region, and do not, therefore, differ in 215 of the 216 villagers. The fact that the GM differences in this cohort are almost entirely in the G3 constant region and the significance of the GM polymorphisms in terms of possible differences in antibody functionality between the susceptible (i.e. 13, 6 containing allotypes) and the less susceptible (i.e. phenotypes not containing 13, 6 allotypes), worth further investigation. In this study, the GM 1,17 5,14 carriers, compared with the carriers of the other phenotypes or the non-carriers of 1,17 5,14 phenotype, had experienced the least number of malaria episodes although the differences were not significant. The significantly raised antibodies in the plasma of the carriers of the GM 1,17 5,13,14,6 phenotype, were mainly the total IgG and IgG2, but not the IgG3 isotype. The levels of IgG4 isotype to all antigens were generally very low, not associated with age and apparently were not increased after acute infection (unpublished data). The allotype-specific variation in the levels of IgG subclasses was antigen-dependent. This finding is supporting and adding to a previous report suggesting inherent ability of individual malaria antigens in antibody class switching [23].

The GM 1,17 5,13,14,6 phenotype was the second most prevalent phenotype in the area. This same phenotype was found to be more dominant in the Massalit, a tribe presumed to be more susceptible to malaria than Fulani [12]. The above-mentioned implication of GM 1,17 5,13,14,6 phenotype, might be due to the GM 6 allotype component. The allotype 6 was detected in half of the Daraweesh inhabitants, while at a global level the GM phenotypes containing GM 6 was recognized only in Africans, but not in other populations [6]. Recently, it was shown that, there was an inverse relationship between carriage of GM 1,17 5,13,14,6 phenotype and occurrence of uncomplicated malaria in Benin [22]. That observation does not contradict this study findings, as in their study all individuals involved in the study were infected with malaria and there were small in age (less than 10 years). Furthermore, in the Benin study, the clinical grouping was based on a single observation, also, the immune profile of the patients was not known. In this study, the above limitations were surmounted. In addition, the diversity that could result from the differences due to ethnicity was overcome by having a single homogenous ethnic study group. The KM allotypes were not statistically associated with malaria susceptibility.

The influence of GM/KM allotypes on antibody response is assumed to be due to alteration of antibody specificity through variable regions [24] or by modification of antibody constant region [25]. The data showed limited effects for the GM/KM phenotypes on the concentration of IgG isotypes. However, there was a tendency for an antigen-dependent pattern of responses. Having examined five (IgG and subclasses) antibody titres to four antigens in the same individual, lack of consistency in levels of any subclass to all examined antigens in any of the GM/KM phenotypes carrier groups, was an evidence for lack of an innate effect for allotypes in levels of subclasses without the influence of the antigens. The antigen-dependent IgGs responses in malaria, are shown to have opposing significances i.e. exposure or protection, in a recent study from Kenya [26].

Recently, it has also been found that, there is a higher prevalence than previously expected, of primary partial immune deficiencies including IgG isotypes and GM allotypes, in apparently healthy individuals [27]. Finally, the influence of GM allotypes (immunoglobulin C-region) in immunity is conventionally thought to be associated with the V-region, as a particular allotype might be in linkage disequilibrium with a particular V- region determinant [28]. Alternatively, GM6 and GM13 allotypes, in this study, could contribute to formation of idiotypes associated with lower immunological responsiveness. On the other hand, the C-region (namely CH2 and CH3 domains) could modify the immunity through its affinity to the FcγR, and there might be certain GM allotype interaction with particular FcγR variant leading to either increased or decreased immune response [28]. A work showing the association between GM allotypes and FcγR polymorphisms and another showing the association between the levels of total IgG and IgG subclasses to different antigens and protection from malaria in the same cohort, are in progress.

## Conclusion

This study shows that, the GM allotypes are involved in susceptibility to uncomplicated malaria. Furthermore, there are indications for involvement of GM and to a lesser extent of KM allotypes in control of total IgG and IgG isotype concentrations, depending on target malaria antigen. The GM 1,17 5,13,14,6 phenotype was associated with higher baseline levels of total IgG and non-cytophilic IgG2. While GM 1,17 5,14 phenotype was associated with least susceptibility and highest baseline IgG3 response. Finally, the allotype GM 6, which is believed to be absent in Europeans was detected in 50% of the study population (Fulani).

## Competing interests

The authors declare that they have no competing interests.

## Authors' contributions

HG, DA and TT planed, established, maintained and supported the Daraweesh nine-years clinical and parasitological data; HG, AN and GE supervised and collected the field samples; AR, NI, MTB, KB, GH and JP were responsible for the laboratory data, HG, DA, TT, MTB, KB, JP and GH, were responsible for the study design, data analysis, results interpretation and paper drafting, all authors contributed to manuscript writing
